# The Comparative Anatomy of Domesticated Animals

**Published:** 1891-11

**Authors:** 


### REVIEWS.

THE COMPARATIVE ANATOMY OF THE DOMESTICATED ANIMALS. By A. Chauveau, revised and enlarged, with the co-operation of S. Arloing; *Second English Edition*, translated and edited by George Fleming, C.B., L.L.D., F.R.C.V.S., etc. with 585 illustrations. D. Appleton and Co., New York, 1891. $7.00.

This much needed second edition of Chauveau's anatomy is dedicated in commemoration of the centenary of the Royal Veterinary College, London and in memory of Charles Vial de Saint Bel. In seventeen years, since the first English edition was printed, the original French has seen three new editions. Considerable amendments, alterations and additions have been made in order to continue this invaluable work, as a complete text book and standard of reference for the veterinary student and practitioner. The anatomy of the ass, mule, rabbit and camel has been added and one hundred and thirty new illustrations aid the text and reference matter. A copious index renders the thousand and odd pages practical and accessible for review work by the student, and surgical reference by the busy practitioner. The translator has adopted the excellent system of having larger type for the more important parts and smaller type for those of less moment.

The veterinarian and comparative anatomist, alike, will find this anatomy an indispensable part of their library.

